# Hop/Sti1 – A Two-Faced Cochaperone Involved in Pattern Recognition Receptor Maturation and Viral Infection

**DOI:** 10.3389/fpls.2017.01754

**Published:** 2017-10-11

**Authors:** Christian E. Lamm, Max. E. Kraner, Jörg Hofmann, Frederik Börnke, Hans-Peter Mock, Uwe Sonnewald

**Affiliations:** ^1^Department of Biology, Division of Biochemistry, Friedrich-Alexander University Erlangen-Nuremberg, Erlangen, Germany; ^2^Leibniz Institute of Vegetable and Ornamental Crops, Großbeeren, Germany; ^3^Leibniz Institute of Plant Genetics and Crop Plant Research, Gatersleben, Germany

**Keywords:** Hop/Sti1 cochaperone, pattern recognition receptor, CERK1, FLS2, pathogen perception, *Nicotiana tabacum* cv. Samsun NN, Potato virus Y

## Abstract

Perception of pathogens by host pattern recognition receptors (PRRs) or R proteins is a prerequisite to promote successful immune responses. The Hsp70/Hsp90 organizing protein Hop/Sti1, a multifunctional cochaperone, has been implicated in the maturation of a receptor-like kinase (RLK) necessary for chitin sensing. However, it remains unknown whether Hop/Sti1 is generally participating in PRR genesis. Using RNA-interference (RNAi), we silenced Hop/Sti1 expression in *Nicotiana tabacum* to gain further insight into the role of the cochaperone in plant defense responses. As expected, transgenic plants do not respond to chitin treatment anymore. In contrast to this, trafficking and functionality of the flagellin PRR FLS2 were unaltered, suggesting a selective involvement of Hop/Sti1 during PRR maturation. Furthermore, Hop/Sti1 was identified as a cellular determinant of Potato virus Y (PVY) symptom development in tobacco, since PVY was able to accumulate to near wild-type level without provoking the usual veinal necrosis phenotype. In addition, typical antiviral host defense responses were suppressed in the transgenic plants. These data suggest that perception of PVY is dependent on Hop/Sti1-mediated receptor maturation, while viral symptoms represent a failing attempt to restrict PVY spread. In addition, Hop/Sti1 colocalized with virus-induced membrane aggregates in wild-type plants. The retention of Hop/Sti1 in potential viral replication complexes suggests a role during viral translation/replication, explaining why RNAi-lines do not exhibit increased susceptibility to PVY. This study provides evidence for a dual role of Hop/Sti1 in PRR maturation and pathogen perception as well as in promoting viral proliferation.

## Introduction

Virtually all forms of life are subjected to a constant threat of pathogen attack. In the course of evolution, animals as well as plants have developed sophisticated mechanisms in order to defend themselves from such attacks, while pathogens have evolved strategies to circumvent these lines of defense in this coevolutionary arms race. One prerequisite for successful defense is recognition of the pathogen. To this end, plants as well as animals are able to sense conserved molecules specific to pathogens known as pathogen- or microbial-associated molecular patterns (PAMPs, MAMPs) by the use of plasma membrane-localized pattern recognition receptors (PRRs) (Nürnberger and Brunner, 2002; Macho and Zipfel, 2014; Zipfel, 2014). In the plant kingdom, the perception of the flagellin-derived peptide flg22 by the leucine-rich repeat (LRR) receptor-like kinase (RLK) FLAGELLIN-SENSING 2 (FLS2) together with co-receptor BRI1-associated receptor kinase 1 (BAK1) is one of the best studied interactions (Felix et al., 1999; Gomez-Gomez and Boller, 2000; Bauer et al., 2001; Chinchilla et al., 2006, 2007; Sun et al., 2013; Liang et al., 2016). Other examples for PAMPs that can be sensed by plant PRRs include the bacterial elongation factor Ef-Tu or the fungal cell wall-component chitin (Kunze et al., 2004; Miya et al., 2007; Shimizu et al., 2010; Liu et al., 2012). Common to all successful interactions of PRRs and PAMPs is the elicitation of the PAMP-triggered immunity (PTI), a multitude of defense responses including the generation of reactive oxygen species (ROS) or expression of pathogenesis-related genes to parry the pathogen attack (Bigeard et al., 2015). To suppress these defense responses, pathogens have evolved effectors, leading to effector-triggered susceptibility (Jones and Dangl, 2006; Dodds and Rathjen, 2010; Macho and Zipfel, 2015). The next tier of the plant immune system is represented by mostly intracellular disease resistance proteins (R proteins), which are able to recognize pathogenic effectors either directly or indirectly by “guarding” other cellular proteins (Dangl and Jones, 2001; Stuart et al., 2013). When activated, R proteins induce a strong and rapid defense response, which may ultimately lead to a local programmed cell death reaction known as hypersensitive response (HR), serving to confine the pathogen infection and thus conferring effector triggered immunity (ETI) (Jones and Dangl, 2006; Cui et al., 2015).

Although these plant immune responses are already well-investigated, some of the underlying mechanisms are just being unraveled. For instance, the cellular processes involved in PRR maturation and trafficking remained largely unresolved until the group of Ko Shimamoto was able to assign the transport of a RLK crucial for chitin-perception in rice, chitin elicitor receptor kinase 1 (OsCERK1), to the chaperone Hsp90 and the cochaperone Hsp70/Hsp90 organizing protein, also known as stress-induced protein 1 (Hop/Sti1) (Chen et al., 2010; Shimizu et al., 2010). This finding was also adopted by Popescu (2012), whose review features both Hop/Sti1 and Hsp90 in a model for a multi-step trafficking pathway from the endoplasmic reticulum (ER) to the plasma membrane for signaling receptors in plants. In general, the function of Hop/Sti1 is to tether Hsp90 to Hsp70, forming a ternary complex (Chen and Smith, 1998). Therefore, the cochaperone, which is conserved across kingdoms, is a substantial part of the protein folding machinery, allowing for client protein transfer from Hsp70 to Hsp90 in a tightly regulated manner (Johnson et al., 1998; Zhang et al., 2003; Alvira et al., 2014; Rohl et al., 2015). Interestingly, the maturation of steroid receptors in mammals was shown to be aided by Hop/Sti1, paralleling the observations of PRR/RLK maturation in rice to some extent (Chen et al., 1996; Dittmar et al., 1996; Morishima et al., 2000; reviewed by Smith, 2004). Apart from this, the cochaperone was also implicated in post-translational protein targeting to yeast mitochondria and to *Arabidopsis thaliana* chloroplasts by preventing premature folding and/or aggregation, thus allowing for import into these organelles (Fellerer et al., 2011; Hoseini et al., 2016).

Unlike most phytopathogenic bacteria and fungi, plant viruses reside inside the cells of their host plants, largely evading detection by plasma membrane-localized PRRs. Therefore, resistance to viruses is predominately conferred by activation of corresponding intracellular R proteins, basically following the long standing gene-for-gene hypothesis (Flor, 1971; Mandadi and Scholthof, 2013). In susceptible plants, viruses are able to spread systemically and cause manifold disease symptoms. Potato virus Y (PVY), a member of the large genus of potyviruses, is able to cause damage in a wide range of economically important crop plants, including *Solanaceae* species such as potato (*Solanum tuberosum*), tobacco (*Nicotiana tabacum*), tomato (*Solanum lycopersicum*), and pepper (*Capsicum* spec.) (Quenouille et al., 2013). Depending on the virus strain, symptoms in potato include stunted growth, vein necrosis, mosaic and deformation of leaves and development of the tuber necrotic ringspot disease (Beczner et al., 1984; Quenouille et al., 2013). In *Nicotiana tabacum* cv. Samsun NN, infections with the necrotic strain of PVY (PVY^N^) cause necrosis in leaf veins and stems, leaf deformations and stunted growth (Singh et al., 2008; Faurez et al., 2012). The initial cause of the development of these symptoms, however, is not yet entirely resolved. Thus, it is unclear whether they are, as suggested by Mandadi and Scholthof (2013), a consequence of a failing HR-like programmed cell death response to restrict viral spread, or whether the ravaging effect of cellular rearrangement induced by viral proteins may be significantly involved in this specific phenotype. Likewise, the cellular mechanisms underlying a potential insufficient defense reaction remain unknown.

In order to gain insight into the defense-related functions of Hop/Sti1, we generated *Nicotiana tabacum* cv. Samsun NN lines stably expressing an RNA-interference construct to silence the expression of the cochaperone. Here, we report that Hop/Sti1, although necessary for maturation of CERK1 (Chen et al., 2010), is not required for targeting or function of FLS2. Furthermore, our study reveals that the cochaperone functions as determinant of PVY^N^ symptom development in tobacco, since Hop/Sti1 RNAi-lines were tolerant to the virus. Despite only moderate reductions in virus spread, typical defense reactions in response to virus infection were absent in transgenic plants, suggesting a defect in PVY^N^ perception. Infected wild-type cells show a colocalization of Hop/Sti1 with ER-derived membrane aggregates, likely representing viral replication complexes. Regarding this localization, we analyzed the integrity of the ER stress-induced unfolded protein response (UPR) in transgenic plants and found it unaltered. Taken together, our study specifies the role of Hop/Sti1 in PRR-maturation and extends its functional range toward host–virus interactions.

## Materials and Methods

### Plant Material and Virus Inoculation

*Nicotiana benthamiana* and *Nicotiana tabacum* cv. Samsun NN were grown in a greenhouse maintaining a 16 h light/8 h darkness photoperiod and temperatures of 25°C and 20°C, respectively. PVY^N^ infections were carried out by mechanical inoculation as described earlier (Herbers et al., 1996).

### Plasmid Constructs

The 35Sp::GFP-NtHop/Sti1 construct was generated using the Gateway^®^ Cloning system (Thermo Fisher Scientific, Waltham, MA, United States): The coding sequence of NtHop/Sti1 was amplified from *Nicotiana tabacum* cv. Samsun NN cDNA by PCR (Oligonucleotide primers: fwGFP-Hop: 5′-CACCGCCGACGAAGCTAAGGC-3′; revGFP-Hop: 5′-CTATCATTGGACAATTCCTGCATTAATCAACTTTTG-3′) and subcloned into pENTR/D-TOPO^®^(Thermo Fisher Scientific) according to the manufacturer’s advice. The final construct was generated using the LR-Clonase^®^ Enzyme Mix (Thermo Fisher Scientific) and the destination vector pK7WGF2 (Karimi et al., 2002).

The plasma membrane-GFP marker as well as the ER-mCherry marker generated by Nelson et al. (2007) were obtained from the Arabidopsis Biological Resource Center^1^ as stock # CD3-1003 and CD3-959, respectively.

The FLS2p::FLS2-3xmyc-mCherry construct was kindly provided by Prof. Silke Robatzek (The Sainsbury Laboratory, Norwich, United Kingdom).

### Generation of NtHop/Sti1-RNAi Lines

A 578 bp NtHop/Sti1 fragment (Nucleotides +1150 to +1728 relative to the ATG codon) was amplified from tobacco leaf cDNA (Oligonucleotide primers: NtHOP-RNAi-for: 5′-CACCTTAACTTGGACAATTCCT-3′; NtHOP-RNAi-rev: 5′-AGAGCAGCAAGAGTATTTCAATC-3′). The resulting amplicon was inserted into the pENTR-D/TOPO^®^ vector (Thermo Fisher Scientific) according to the manufacturer’s instructions. The fragment was subsequently recombined into the destination vector pK7GWIWG2(II) (Karimi et al., 2002) using LR-Clonase^®^ Enzyme Mix (Thermo Fisher Scientific). The resulting plasmid was transformed into *Agrobacterium tumefaciens* strain C58C1 harboring pGV2260 (Deblaere et al., 1985). The transformation of tobacco was carried out following Horsch et al. (1985).

### Heterologous Expression of NtHop/Sti1 in *E. coli*

The NtHop/Sti1 open reading frame was amplified from a tobacco cDNA template using appropriate oligonucleotide primers (NtHOP-pQE32-for: 5′-AATCCCGGGGCCGACGAAGCTAAG-3′; NtHOP-pQE32-rev: 5′-CGCGAAGCTTTTATTTAACTTGGACAATTCC-3′) which create a SmaI site at the 5′ end of the amplicon and a HindIII site at its 3′ end. The amplicon was then cloned into the pCR2.1 vector (Invitrogen, now Thermo Fisher Scientific), and the construct validated by sequencing. The NtHop/Sti1 coding sequence was excised by digestion with SmaI and HindIII, directionally cloned into the pQE32 expression vector (Qiagen, Hilden, Germany), and introduced into *E. coli* strain XL1 Blue (Bullock et al., 1987). An aliquot of an overnight *E. coli* culture grown at 37°C in LB medium containing 100 μg ml^-1^ ampicillin was used to inoculate 50 ml fresh LB medium containing ampicillin and grown at 37°C to an OD_600_ of 0.5–0.6. IPTG (isopropylthio-ß-galactoside) was added to a final concentration of 1 mM to induce the expression of the HIS::NtHop/Sti1 fusion protein and the culture was allowed to grow for an additional 2 h at 37°C. The cells were then pelleted by centrifugation and re-suspended in three volumes of 50 mM NaH_2_PO_4_, 300 mM NaCl, 10 mM imidazole, pH 8.0 containing HP proteinase inhibitor (Serva, Heidelberg, Germany). After 30 min of incubation in the presence of 1 mg ml^-1^ lysozyme, the cells were lysed by sonication on ice and the lysate was clarified by centrifugation (16,000 × *g*, 30 min, 4°C). Protein concentration in the lysate was determined by the Bradford method.

### Affinity Purification of the Recombinant NtHop/Sti1 and Generation of an Antibody

The purification of the recombinant NtHop/Sti1 protein was carried out using a HisTrap Kit (GE Healthcare, Little Chalfont, United Kingdom). The lysate was loaded on an equilibrated HiTrap Chelating HP 1 ml column at a flow rate of 0.25 ml min^-1^. Non-specifically bound proteins were eluted by washing with 20 ml of 50 mM NaH_2_PO_4_, 300 mM NaCl, 20 mM imidazole, pH 8.0. The target proteins were then eluted from the column by the passage of 7 ml 50 mM NaH_2_PO_4_, 300 mM NaCl, 500 mM imidazole, pH 8.0. The purity of the recombinant NtHop/Sti1 protein was confirmed by SDS-PAGE (Laemmli, 1970).

Following the immunization of rabbits with purified, recombinant NtHop/Sti1, antisera were affinity purified on a 5 ml HiTrap-NHS column using the ÄktaExplorer System (GE Healthcare). Isopropanol was first removed from the column by washing three times in ice cold 1 mM HCl and the column was then loaded with 2 mg recombinant NtHop/Sti1 for 30 min at room temperature. Non-specifically bound proteins were removed by washing with 25 ml of 140 mM NaCl, 10 mM KCl, 6.4 mM Na_2_HPO_4_, 2 mM KH_2_PO_4_. Washing and deactivation of the column were achieved by alternate incubation with 0.5 M ethanolamine, 0.5 M NaCl, pH 8.3 and 0.1 M acetate, 0.5 M NaCl, pH 4. The antiserum was desalted by passing through a PD-10 column (GE Healthcare) and then loaded onto the equilibrated HiTrap-NHS column at a flow rate of 0.3 ml min^-1^. After the removal of non-specifically bound proteins (with 40 ml of 0.01 M Tris, 1 M NaCl, pH 7.5), the antibodies were eluted with 0.1 M glycine, pH 2.8 and neutralized by the addition of Tris-HCl pH 8.0. The purity was confirmed by immunoblot analysis.

### Protein PAGE and Western Blot Analysis

To obtain total protein extract, two leaf disks (Diameter ∼9 mm) were frozen in liquid nitrogen, ground in 140 μl extraction buffer (90 mM Tris HCl pH 6.8, 20% glycerol, 2% SDS, 0.02% bromophenol blue, 100 mM DTT), boiled for 10 min at 95°C and centrifuged for 3 min at 15,000 × *g*. Supernatant was loaded on a Bis/Tris gel containing 12% acrylamide for electrophoresis. Proteins were transferred onto a nitrocellulose membrane, which afterward was blocked for at least 1 h in blocking buffer [20 mM Tris HCl, 500 mM NaCl, 0.1% (v/v) Tween20, 5% (w/v) milk powder]. Detection was carried out using suitable primary antibodies as indicated and horseradish peroxidase conjugated secondary antibodies, followed by enhanced chemiluminescence detection.

Nitrocellulose membrane stainings were conducted using a solution of 0.5% (w/v) Ponceau S in 1% acetic acid. Membranes were incubated for 2 min in the staining solution and subsequently destained in distilled water.

### Relative Measurement of Reactive Oxygen Species

The relative amount of ROS was measured in response to treatment of leaf disks (Diameter ∼4 mm, three biological replicates per line) with 1 μM flg22 or 20 μg ml^-1^ hexa-*N*-acetyl-chitohexaose according to the bioassay established by Smith and Heese (2014).

### Transient Expression in *Nicotiana* spec. and Confocal Laser Scanning Microscopy

*Agrobacterium tumefaciens* (strain C58C1)-mediated transient expression in *Nicotiana* spec. was carried out as described (Deblaere et al., 1985; Sparkes et al., 2006). Microscopy was performed using a Leica TCS SP5 II confocal laser scanning microscope (Leica Microsystems, Wetzlar, Germany) as described earlier (Lamm et al., 2016).

### Double Antibody Sandwich Enzyme-Linked Immunosorbent Assay

Enzyme-linked immunosorbent assays were performed using a PVY CP ELISA Kit (Bioreba, Reinach, Switzerland) according to the manufacturer’s advice. In short, one *N. tabacum* SNN leaf disk (Diameter ∼9 mm) obtained from a systemically infected leaf (five leaves above inoculation site) was ground in 600 μl of the provided extraction buffer containing cOmplete ULTRA Tablets (Roche, Penzberg, Germany). Following 1 min of centrifugation at 15,000 × *g*, the supernatant was used for the generation of serial dilutions. Protein dilutions were incubated overnight in Nunc MaxiSorp microtiter plates (Thermo Fisher Scientific) coated with a cocktail of PVY CP monoclonal antibodies raised against different PVY strains. Following three washing steps with PBS-Tween with a Bio-Tek ELx50 plate washer (Bio-Tek, Minooski, VT, United States), a solution of PVY CP monoclonal antibodies conjugated with alkaline phosphatase was added to the wells. After 4 h of incubation at room temperature, the plates were washed four times as described above, and 200 μl of a 1 mg ml^-1^ solution of *p*-nitrophenyl phosphate (pNPP) was added. The reaction was stopped after 20 min of incubation in the dark via addition of 100 μl 3 M NaOH. Finally, absorbance was measured at 405 nm using an EL808 plate reader (Bio-Tek). Data evaluation was carried out with eight, nine, seven, or ten considered biological replicates for wild-type, Hop/RNAi line #17, #59 and #61, respectively.

### Grafting of *Nicotiana tabacum* Plants

For grafting of non-infected scions on PVY-infected rootstocks, designated *Nicotiana tabacum* stocks were mechanically inoculated with PVY^N^ as indicated above. As soon as early infection symptoms could be observed in wild-type plants, the stems were cut horizontally and, using a razorblade, a V-shaped slit was introduced into the stem. The designated non-infected scion was cut to fit the slit with a fresh razorblade. After joining both stock and scion, the graft was sealed with parafilm and several leaves were detached to ensure proper water supply. For the first week after grafting, plants were kept in a plastic film-covered housing in order to maintain high humidity.

### Generation of cDNA, RT-PCR, and Quantitative Real-time PCR

RNA was isolated from wild-type *Nicotiana tabacum* plants and from *Nt*Hop/Sti1-silenced plants, both PVY-infected and uninfected, as described before (Logemann et al., 1987). The obtained RNA was then used to generate cDNA, using the Bio-Rad iScript cDNA synthesis kit (Bio-Rad) for analysis of PR-gene expression or the RevertAid H Minus Reverse Transcriptase (Thermo Fisher Scientific) for analysis of bZIP60 splicing status, PVY genome detection and analysis of Hop/Sti1 expression.

For PVY RNA detection, cDNA was amplified using Taq polymerase and oligonucleotide primers specific for the viral capsid protein (CPfw: 5′-CGGAGTTTGGGTTATGATGG-3′; CPrev: 5′-ACGCTTCTGCAACATCTGAG-3′). The band intensities in the successional agarose gel were determined using the ImageJ software. Quantitative real-time PCR analyses were performed using specific oligonucleotide primers for *Nicotiana tabacum* PR-1 and PR-2 (PR1-for: 5′-TTGCCGTGCCCAAAATTCTC-3′; PR1-rev: 5′-ACAATCTGCAGCCAATTGGG-3′; PR2-for: 5′-TGCTCCTGCCATGCAAAATG-3′; PR2-rev: 5′-ATACTATCTTTGGGCGGGTTGG-3′), Hop/Sti1 (Hop-fw: 5′-AGACGAAGAACGCGAGAAAG-3′; Hop-rev: 5′-TATATGCCCTCGGGTCTTTG-3′) or spliced bZIP60 (bZIP60F: 5′-GCAGAAAATCAAGCTTTGCGCT-3′; bZIP60SR: 5′-AGGGAACCCAACAGCAGACT-3′) as reported earlier (Gaguancela et al., 2016). For normalization of target gene expression, the expression of either actin or ubiquitin was determined (Act-for: 5′-GCTGGATTTGCTGGTGATG-3′; Act-rev: 5′-TCCTTCTGTCCCATTCCGAC-3′; UBQ10fw: 5′-AGATCCAGGACAAGGAAGGTATTC-3′; UBQ10rev: 5′-CGCAGGACCAAGTGAAGAGTAG-3′). As a reference dye, SYBR green was used (Brilliant II SYBR^®^ MM or Brilliant III SYBR^®^ MM, Agilent, Santa Clara, CA, United States). The reactions were performed in triplicates in Agilent MX3000P or AriaMx real-time thermocyclers. Data evaluation was performed either by the Stratagene MxPro software (Agilent) or (when the AriaMx thermocycler was used) according to Livak and Schmittgen (2001).

### Extraction of Salicylic Acid and HPLC Analysis

Salicylic acid (SA) was extracted as reported earlier (Nawrath and Metraux, 1999). To each sample 250 ng of ortho-anisic acid were added as internal standard to which the measured values were corrected, as described before (Meuwly and Metraux, 1993). For each line, three to four biological replicates were included in the evaluation. HPLC separation of SA and *o*-anisic acid was performed on a Dionex Summit system (P680, ASI-100, TCC-100, RF-2000, Thermo Fisher) equipped with a Phenomenex Luna Security Guard C18 column (4.0 mm × 3.0 mm, Phenomenex, Torrance, CA, United States) followed by a 5-μm Luna C18(2) reverse-phase column (250 mm × 4.6 mm, Phenomenex) as described by Voll et al. (2012).

### Measurement of Electrical Conductivity

As a proxy for cell death in response to tunicamycin treatment of *N. tabacum* leaf disks (Diameter ∼9 mm), the increasing electrical conductivity caused by leakage of ions into the floating medium was measured. In short, leaf disks were floated abaxial side down on 1 ml H_2_O in a 24-well plate for 1–2 h. Subsequently, the water was discarded and replaced by either 1 ml of 10 μg ml^-1^ tunicamycin in 0.4% dimethyl sulfoxide (DMSO) or 1 ml 0.4% DMSO as control. At each timepoint, 140 μl of the floating medium was pipetted on the electrode of a conductivity meter (LAQUAtwin, Horiba Scientific, Bensheim, Germany) for measurement and afterward returned into its well. Data analysis was carried out with four to six biological replicates.

### Statistical Analyses

Bonferroni-corrected student’s *t*-tests were carried out in Microsoft Excel 2010 (Microsoft, Redmond, WA, United States), while analysis of variance (ANOVA) followed by Tukey’s Honestly Significant Difference test was performed using IBM SPSS Statistics 24 (IBM, Armonk, NY, United States).

## Results

### Generation of Hop/Sti1-Silenced Transgenic *Nicotiana tabacum* Plants

**FIGURE 1:**
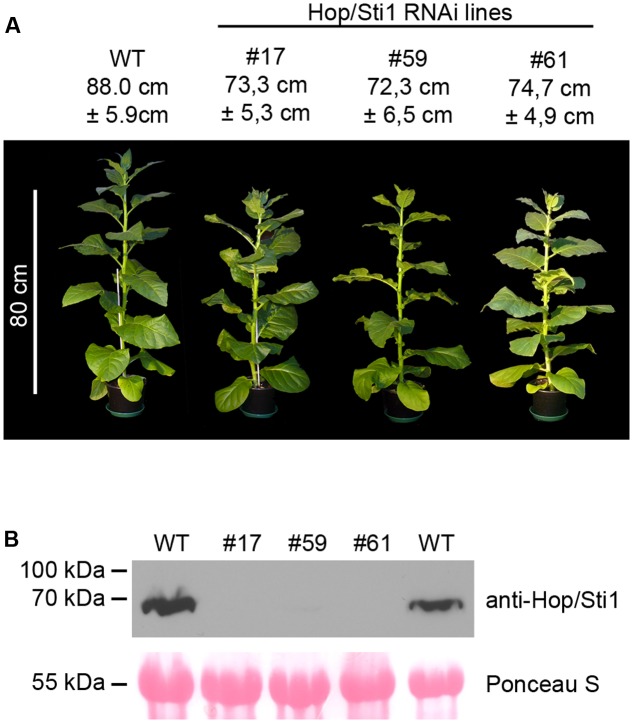
Characterization of Hop/Sti1-RNAi plants. **(A)** Morphology of wild-type (WT) *Nicotiana tabacum* cv. Samsun NN plant and Hop/Sti1-RNAi plants from three independent lines. Height of the plants is given as average plus/minus standard deviation, calculated from ten individual plants per line. Difference between wild-type and transgenic plants is significant (Bonferroni-corrected student’s *t*-test, *p*-values of 4.51E–05, 7.14E–05 and 1.09E–04 for lines #17, #59, and #61, respectively) **(B)** Western blot analysis of Hop/Sti1 protein level in wild-type and Hop/Sti1-RNAi lines using an anti-Hop/Sti1 antibody. Hop/Sti1 is detectable in wild-type plants, but not in the transgenic lines. Ponceau S staining confirms equal loading of the gel.

The expression of the cochaperone Hop/Sti1 in *Nicotiana tabacum* cv. Samsun NN plants was downregulated by posttranscriptional gene silencing using stable expression of a hairpin RNA interference (RNAi) construct. The efficiency of Hop/Sti1 silencing in T_0_-plants was tested by Western blotting using affinity purified polyclonal antibody raised against Hop/Sti1. Out of 78 regenerated plants, three independent lines (#17, #59, and #61) with almost no detectable Hop/Sti1 protein in leaves were selected for further analysis. Compared to wild-type, T_1_-offspring of these lines showed no drastic growth phenotype but a mild, yet significant growth retardation (**Figure 1A**, Bonferroni-corrected student’s *t*-test, *p*-values of 4.51E-05, 7.14E-05, and 1.09E-04 for lines #17, #59, and #61, respectively). To confirm the RNAi-mediated absence of Hop/Sti1 in the T_1_-generation, quantitative real-time PCR analysis and Western blotting was conducted. In agreement with a downregulation of Hop/Sti1-mRNA (Supplementary Figure S1), crude protein extracts from leaves of transgenic plants showed no or almost no detectable Hop/Sti1 protein, while a strong signal could be obtained in extracts from wild-type plants (**Figure 1B**). This indicates that Hop/Sti1 is efficiently silenced in the three selected lines, offering an ideal basis for further analysis.

### Hop/Sti1-Silencing Impairs Maturation of Receptor-Like Kinase CERK1, But Not FLS2

**FIGURE 2:**
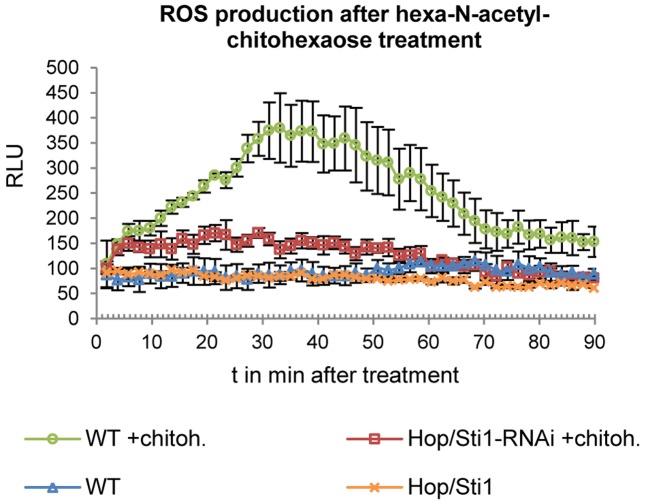
Detection of ROS production in response to hexa-*N*-acetyl-chitohexaose treatment in wild-type plants and in Hop/Sti1-RNAi line #61. Luminescence is given in relative luminescence units (RLU), error bars represent the standard error.

In rice, Chen et al. (2010) were able to show the crucial role of Hop/Sti1 in the maturation and trafficking of the *N*-acetylchitooligosaccharide-responsive RLK OsCERK1. The group could show that cell cultures not expressing Hop/Sti1 showed greatly decreased and/or delayed expression of defense-related genes when treated with hexa-*N*-acetylchitohexaose. Thus, we employed a similar approach to analyze whether Hop/Sti1-silenced tobacco plants behave in a comparable manner. To this end, we measured hexa-*N*-acetylchitohexaose-induced accumulation of ROS in leaf disks by means of a luminol/horseradish peroxidase-based bioassay (Smith and Heese, 2014). In agreement with results obtained in rice, wild-type control samples exhibited increased ROS-levels when stimulated with the oligosaccharide. In contrast to this, samples derived from the transgenic line #61 showed no comparable increase in ROS-production, confirming both the functional integrity of the RNAi-line and the finding of Chen et al. (2010) in tobacco (**Figure 2**).

**FIGURE 3:**
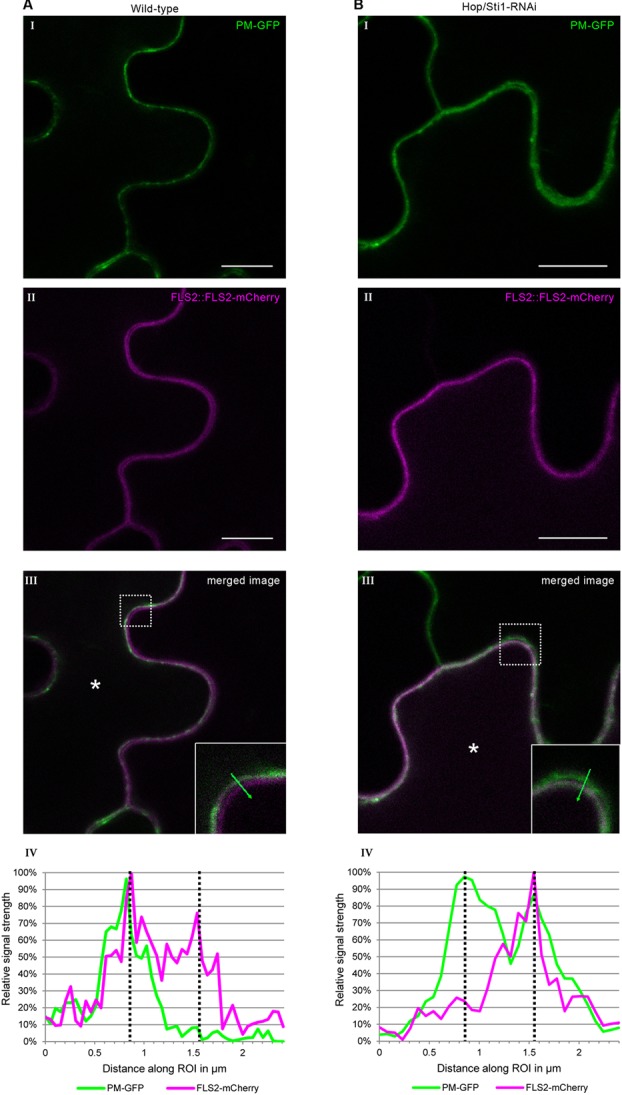
Assessment of FLS2 plasma membrane-targeting using confocal laser scanning microscopy. Coexpression of FLS2-mCherry under control of its own promoter with a plasma membrane-GFP marker. **(A)** In wild-type plants. **(B)** In Hop/Sti1-RNAi line #61. (I) Plasma membrane-GFP marker. (II) FLS2-mCherry. (III) Merged channels. The dotted box is magnified in the lower right corner, an asterisk marks a coexpressing cell. Scale bars represent 10 μm. (IV) A diagram of the fluorescence signal intensities along the region of interest (ROI, green arrow) shown in the inlay in panel III. Dotted lines indicate the intensity maxima representing the plasma membranes of two neighboring cells.

**FIGURE 4:**
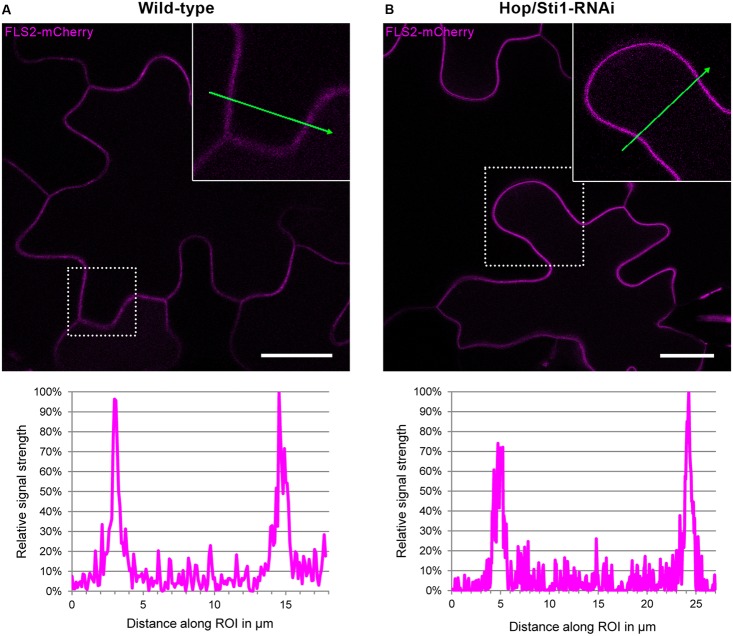
Assessment of potential FLS2-mistargeting in Hop/Sti1-RNAi lines. **(A)** Wild-type *Nicotiana tabacum* transiently expressing FLS2-mCherry, fluorescence signal intensities measured along the ROI (green arrow) are depicted in the diagram below, showing maxima at the plasma membranes and lower basal signal in the cytoplasmic areas. **(B)** Hop/Sti1-RNAi plants transiently expressing FLS2-mCherrry, fluorescence signal intensities depicted analogous to **(A)**. Scale bars represent 20 μm.

**FIGURE 5:**
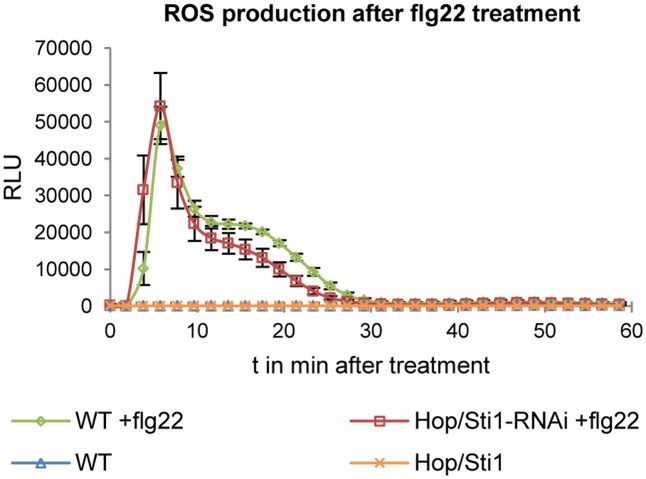
Detection of ROS production in response to flg22 treatment in wild-type plants and in Hop/Sti1-RNAi line #61. Luminescence is given in relative luminescence units (RLU), error bars represent the standard error.

Since the study in rice evaluated the lysine-motif RLK OsCERK1 only, we wondered whether the cochaperone Hop/Sti1 might be generally involved in the trafficking of PRRs to the plasma membrane. In order to test this hypothesis, we analyzed the targeting of the well described leucine-rich repeat RLK FLS2 by confocal laser scanning microscopy (CLSM). *Arabidopsis thaliana* FLS2 tagged with mCherry and under control of its native promoter was expressed transiently in both wild-type *Nicotiana tabacum* cv. Samsun NN plants and Hop/Sti1-RNAi line #61 together with a plasma membrane marker tagged with GFP. Proper FLS2-targeting could be observed in wild-type plants showing overlapping fluorescence signals at the plasma membrane of coexpressing cells (**Figure 3A**). The same result was found when FLS2-mCherry and the plasma membrane marker were expressed in Hop/Sti1-silenced leaves (**Figure 3B**). Furthermore, the ratio of plasma membrane-localized FLS2-mCherry-signal to cytoplasmic signal was found to be comparable in both wild-type and RNAi-line (**Figure 4**), confirming that FLS2 is not mistargeted in the transgenic plants. Both findings indicate that the cochaperone is not, as was shown for OsCERK1, crucial for the transport of FLS2 to the plasma membrane. In addition to this, we examined the functionality of FLS2 in wild-type and Hop/Sti1-RNAi plants with respect to their ability to perceive the FLS2 elicitor, the flagellin-peptide flg22. Again the previously used ROS-bioassay was utilized as a proxy for successful stimulation of the PRR. Thus, we observed a positive and comparable oxidative burst reaction for both wild-type and the transgenic line #61 (**Figure 5**), showing that FLS2 is still functional and, unlike CERK1, Hop/Sti1-independent.

### Hop/Sti1-Silencing Renders *Nicotiana tabacum* Tolerant to Potato Virus Y

**FIGURE 6:**
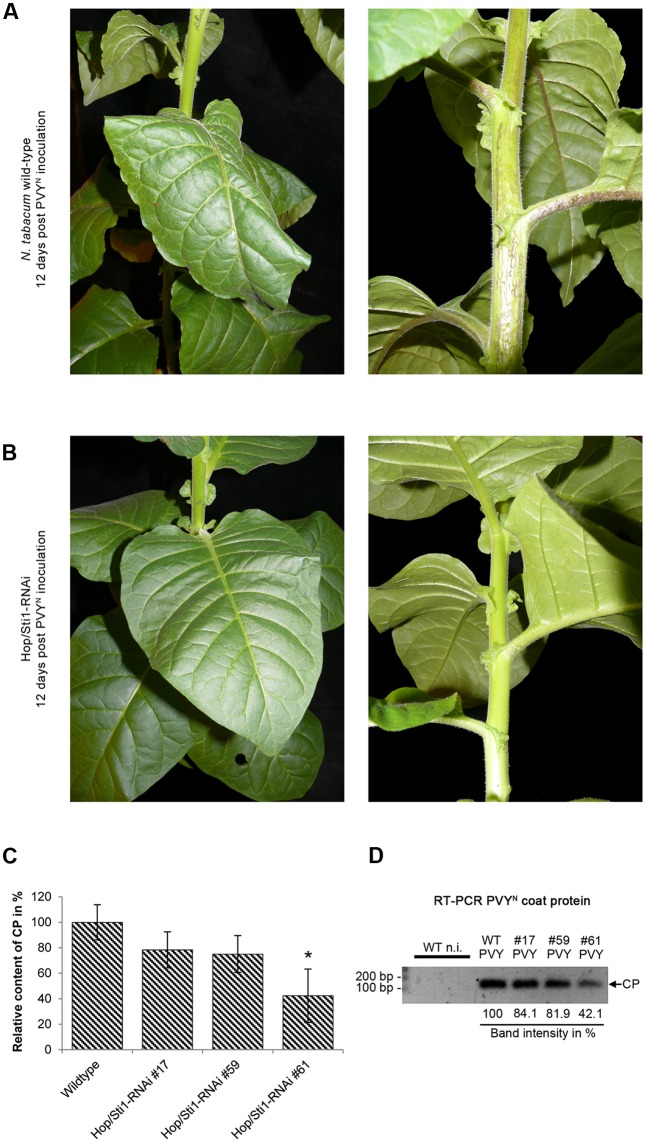
Hop/Sti1-RNAi plants are PVY^N^-tolerant. **(A)** *Nicotiana tabacum* cv. Samsun NN wild-type plant infected by PVY^N^. Twelve days after mechanical inoculation, plants show severe infection symptoms including leaf deformation (left) and necrosis of stem and leaf veins (right). **(B)** Hop/Sti1-RNAi plants infected with PVY^N^. Transgenic plants look healthy and do not show typical disease symptoms 12 days after inoculation. **(C)** PVY-CP ELISA. PVY^N^ titer was determined in infected wild-type and Hop/Sti1-RNAi plants. An asterisk indicates significance (Bonferroni-corrected student’s *t*-test, *p*-value: 1.89E– 02). Error bars represent the standard error. **(D)** Accumulation of viral RNA in infected wild-type and Hop/Sti1-RNAi plants. RT-PCR was used to amplify a 122-basepair fragment of the viral RNA encoding the coat protein (depicted by an arrow labeled CP). The amplicon could be detected in infected wild-type and transgenic plants both, but not in non-infected control plants. Values below depict the respective band intensities relative to the infected wild-type control.

With the silencing of Hop/Sti1, we intended to interrupt the functional conjunction of Hsp70 and Hsp90. Apart from their involvement in plant immune responses, both chaperones are involved in a variety of other cellular processes and plant viruses rely on the chaperone system for their translation, replication, and movement (reviewed by Verchot, 2012). On the other hand, cellular chaperones are also involved in the induction of antiviral immune responses (Mandadi and Scholthof, 2013). Based on this, and since Hop/Sti1 seemed to be required for transport and maturation of RLKs in a selective manner, we raised the question whether the silencing of the cochaperone might also have an impact on processes related to the perception of plant viruses. To investigate this matter, we inoculated both wild-type and Hop/Sti1-RNAi *Nicotiana tabacum* plants with the necrotic strain of Potato virus Y (PVY^N^, type species of the genus *Potyvirus*). Typical symptoms in the interaction between PVY^N^ and *Nicotiana tabacum* cv. Samsun NN include necrosis of stem and leaf veins, leading to leaf deformation (**Figure 6A**, 12 days post-inoculation). In contrast to this, tobacco plants silenced for Hop/Sti1 did not develop such symptoms at any given point of the infection (**Figure 6B**, 12 days post-inoculation). Despite this lack of typical disease symptoms, an ELISA test revealed that the virus accumulation in systemically infected leaves was only slightly decreased compared to wild-type plants (**Figure 6C** and as boxplot: Supplementary Figure S2). From the three independent lines, only line #61 showed a significant reduction of the viral titer (Bonferroni-corrected student’s *t*-test, *p*-value: 1.89E-02). This finding could also be verified by examination of viral RNA accumulation in the same systemically infected leaves by reverse-transcription PCR (**Figure 6D**). Thus, viral RNA could be detected at a level, which was comparable to the wild-type control in two of three independent lines and correlated with the results from the ELISA. Considering a possible abrogation of Hop/Sti1-silencing by viral silencing-suppression, we monitored the expression of the cochaperone by Western blotting. Still, Hop/Sti1 was barely detectable in the transgenic lines, confirming the integrity of the silencing also in PVY^N^-infected plants (Supplementary Figure S3). Taken together, this indicates that Hop/Sti1 is a determinant of PVY^N^ symptom development in *Nicotiana tabacum*.

### Hop/Sti1-Silencing Does Not Affect PVY^N^ Long-Distance Movement and Infectivity

**FIGURE 7:**
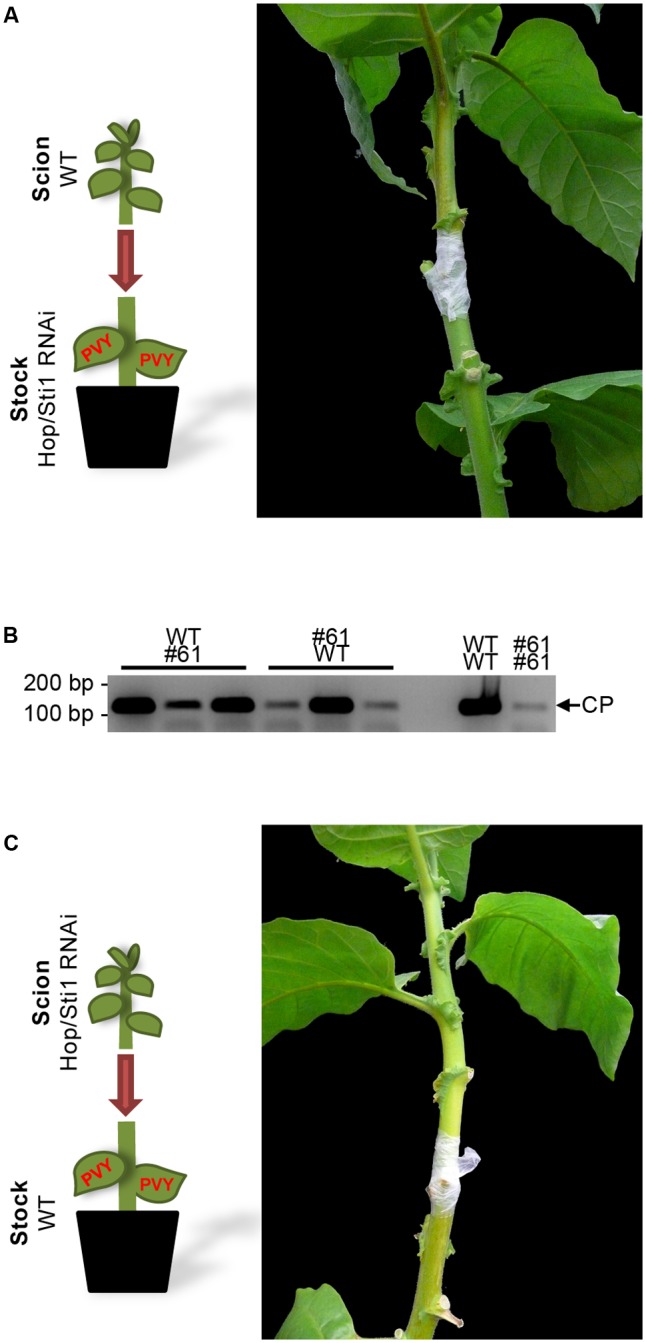
Reciprocal grafting of wild-type and Hop/Sti1-RNAi plants. **(A)** A wild-type scion was grafted on a PVY^N^-infected Hop/Sti1-RNAi rootstock. Two weeks after grafting, the wild-type type scion displayed infection symptoms. **(B)** Accumulation of viral RNA in scions of grafted wild-type and Hop/Sti1-RNAi plants, samples taken 2 weeks after grafting. RT-PCR was used to amplify a 122-basepair fragment of the viral RNA encoding the coat protein (depicted by an arrow labeled CP). Grafting combinations are labeled as scion over infected rootstock. Viral RNA could be detected in all tested mixed grafts (left) as well as in control plants (right, wild-type scion on infected wild-type rootstock, analogous for transgenic line #61). **(C)** Hop/Sti1-RNAi scion grafted on infected wild-type rootstock. Two weeks after grafting, the transgenic scion was still symptom-free, although viral RNA was detectable (compare to **B**).

To investigate whether virus infectivity or long-distance movement through the vascular tissue was impeded by the knockdown of Hop/Sti1, grafting experiments were conducted. Firstly, non-infected wild-type scions were grafted on PVY^N^-infected Hop/Sti1-RNAi rootstocks. As expected, transgenic rootstocks did not show viral symptoms 2 weeks after grafting, while severe symptoms of viral presence could be observed on the wild-type scions (**Figure 7A**). In addition, reverse-transcription PCR was performed to detect viral RNA in systemically infected leaves of the scion. The viral genome could be detected in three individual scions at varying levels that were comparable to control grafts (**Figure 7B**). The observed variation was likely caused by non-synchronized functional symplasmic fusion of scions and rootstocks resulting in differences in viral spreading. Presence of the viral genome in the scions proves that (I) PVY^N^ derived from the transgenic rootstocks is still infectious and (II) systemic virus spreading is not affected by the silencing of the cochaperone.

### PVY^N^ Symptom Development Is Not Mediated by a Systemic Signal and May Not Be Restored by Grafting on Wild-type Rootstock

Next, non-infected transgenic scions were grafted on PVY^N^-infected wild-type rootstocks. While the rootstocks exhibited severe symptoms of PVY^N^ infection, the transgenic scions were symptom-free 2 weeks after grafting (**Figure 7C**). Furthermore, semi-quantitative reverse-transcription PCR confirmed the presence of the viral genome in the Hop/Sti1-RNAi scions (**Figure 7B**). This indicates that the development of PVY^N^ symptoms may not be induced by systemic signaling, thus may not be restored by grafting on an infected wild-type rootstock. Hence, suppression of viral symptoms in Hop/Sti1-RNAi lines has to be ascribed to a defect in a local mechanism.

### Typical Defense Responses after PVY^N^ Infection Are Impaired in Hop/Sti1-Silenced Plants

**FIGURE 8:**
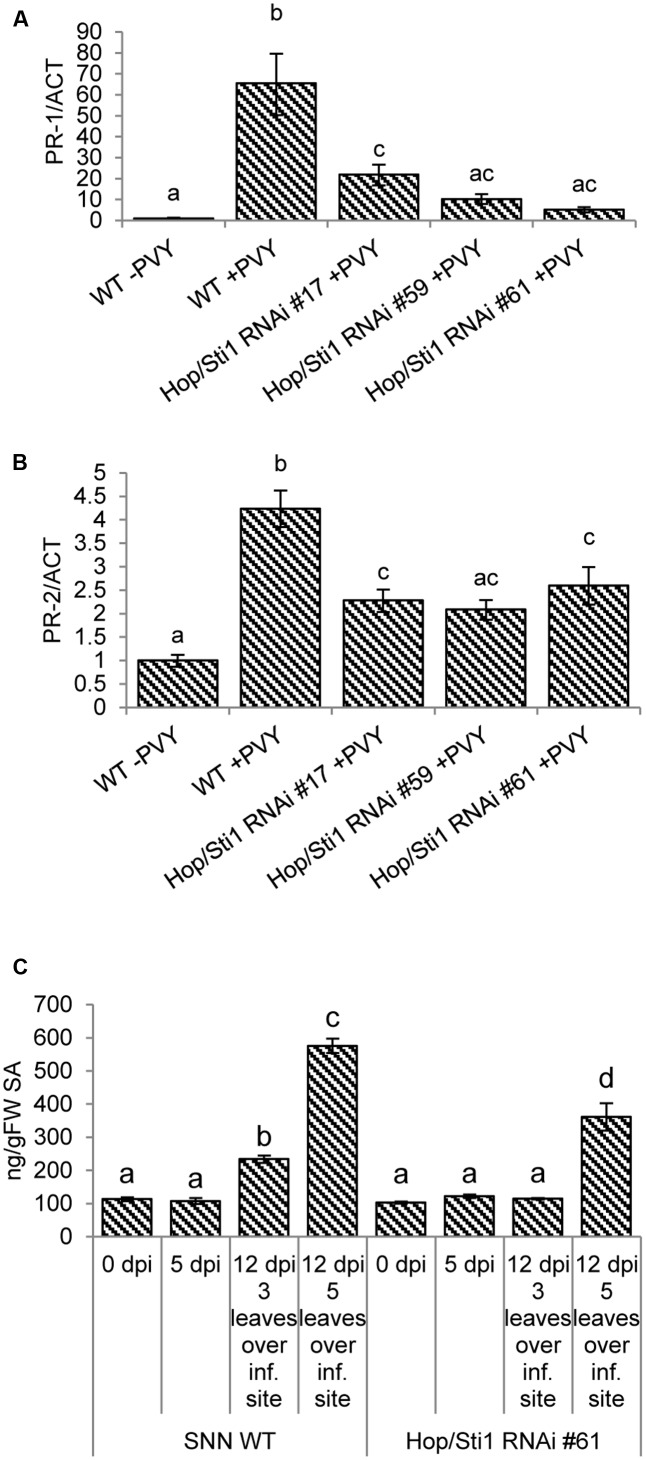
Impaired immune responses after PVY^N^ inoculation in Hop/Sti1-RNAi plants. **(A)** PR-1 expression was analyzed by quantitative real-time PCR. The expression increased significantly (ANOVA, *p*-value: 1.2E–05) in samples derived from systemically infected wild-type leaves compared to non-infected samples. PR-1 expression in infected Hop/Sti1-RNAi lines was in all cases significantly (ANOVA, *p*-values of 3.9E–04, 4.9E–05, 2.2E–05 for lines #17, #59, and #61, respectively) lower than in infected wild-type plants. Error bars represent the standard error. Different letters indicate statistically significant differences (ANOVA, *p* < 0.05). **(B)** PR-2 expression was analyzed by quantitative real-time PCR. Infection of wild-type plants with PVY^N^ led to a significant induction of PR-2 expression (ANOVA, *p*-value: 4.3E–05). Infected transgenic plants again showed a significantly (ANOVA, *p*-values of 2.6E–03, 1.3E–03, and 7.2E–03 for lines #17, #59, and #61, respectively) lower PR-2 expression than infected wild-type plants. Error bars represent the standard error. Different letters indicate statistically significant differences (ANOVA, *p* < 0.05). **(C)** Content of free salicylic acid (SA). In wild-type plants, SA levels increased significantly after 12 days three (ANOVA, *p*-value: 1.8E–03) and five (ANOVA, *p*-value: 2.3E–12) leaves over the initial inoculation site. At the same time, SA content in transgenic plants was not significantly altered three leaves over the inoculation site. Five leaves over the inoculation site, SA levels were significantly (ANOVA, *p*-value: 6.2E–07) lower compared to wild-type. Error bars represent the standard error. Different letters indicate statistically significant differences (ANOVA, *p* < 0.05).

Although susceptible to PVY^N^, wild-type tobacco is still responding to the viral intrusion by induction of basal defense mechanisms such as pathogenesis-related (PR) gene expression, SA mobilization and the production of ROS (reviewed, e.g., by Mandadi and Scholthof, 2013). Given the selective role of Hop/Sti1 in the maturation of PRRs, we hypothesized that in case of PVY^N^ infection of the transgenic plants, the perception of the pathogen may be impaired, leading to a lack of defense response induction, which would otherwise be manifested as disease symptoms. Hence, we investigated the responsiveness of PR-genes and the accumulation of SA in both wild-type and knockdown plants following PVY^N^ infection. In the case of the PR-genes, relative expression levels of PR-1 as well as PR-2 were investigated by quantitative real-time PCR in systemically infected leaves 12 days after inoculation. Compared to non-infected control plants, PR-1 expression in wild-type tobacco was induced approximately 66-fold in response to the viral infection (**Figure 8A**). However, the transgenic plants exhibited a significantly lower induction of the PR-1 gene (5- to 22-fold, analysis of variance: Supplementary Table S1) in comparison. Although not as pronounced as for PR-1, PR-2 expression was also strongly induced in wild-type plants, whereas Hop/Sti1-silenced plants showed a significantly lower induction of PR-2 expression upon virus infection, compared to the control (**Figure 8B**, analysis of variance: Supplementary Table S2). For measurement of free SA content, samples were taken from the third leaf over the PVY^N^ inoculation site prior to, as well as 5 and 12 days after inoculation. At the late time point, additional samples were collected from the fifth leaf over the inoculation site since the symptoms of viral infection in wild-type plants were strongest in this leaf. As shown in **Figure 8C**, the content of SA in wild-type plants stayed constant during the first 5 days, yet rose significantly at day 12 after inoculation (analysis of variance: Supplementary Table S3). In addition, the sample derived from the leaf exhibiting stronger disease symptoms contained an even higher amount of SA. In contrast to this, the SA levels in the transgenic lines stayed constant at all time points in the third leaf over the inoculation site. An increase could only be observed in the samples derived from the fifth leaf, however, to a significantly lower extent compared to the control. Altogether, these data suggest a reduced responsiveness of basal immune mechanisms in the Hop/Sti1-RNAi plants upon PVY^N^ infection, possibly caused by the failure to recognize the virus. At this point it is important to note that, in spite of the impaired basal defense, the PVY^N^ titer in the transgenic lines was not increased but slightly reduced as seen in the previous experiments.

### GFP-Hop/Sti1 Colocalizes with ER-Derived Viral Structures in *Nicotiana benthamiana*

**FIGURE 9:**
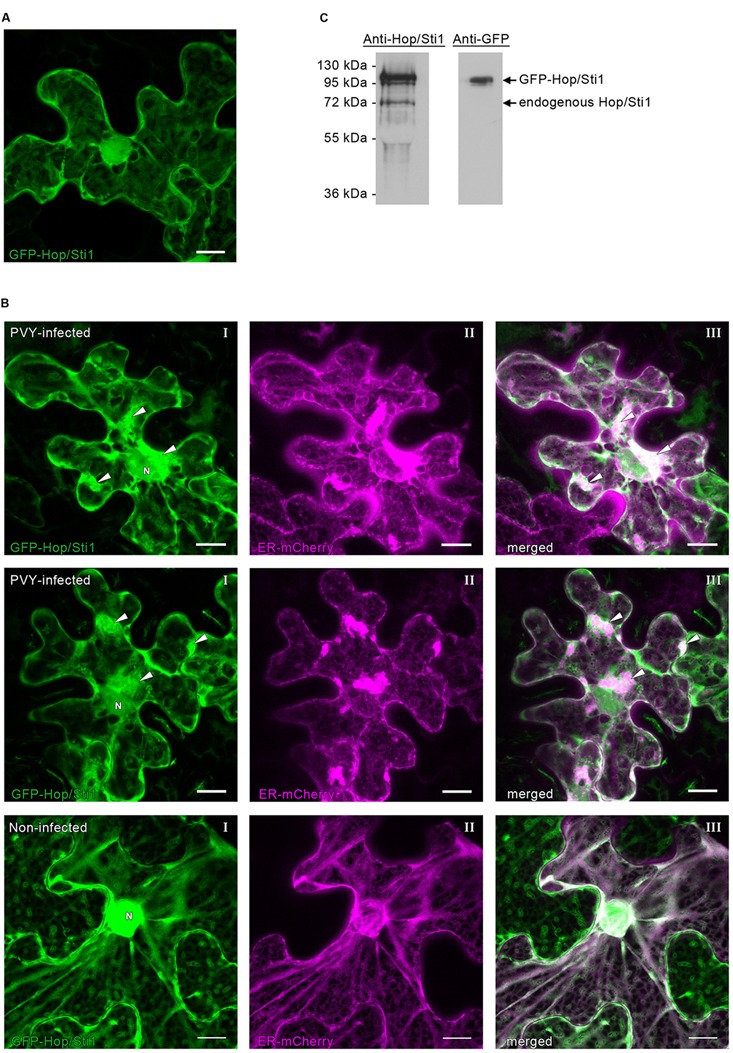
Subcellular distribution of GFP-Hop/Sti1 in *Nicotiana benthamiana*. **(A)** GFP-Hop/Sti1 localized to the cytoplasm, ER, and nucleus. Maximum projection of a confocal Z-stack, scale bar represents 10 μm. **(B)** Detection of GFP-Hop/Sti1 by Western blotting using anti-Hop/Sti1- or anti-GFP-antibodies. With the anti-Hop/Sti1 antibody, both the endogenous protein and the GFP-fusion protein could be detected as indicated by arrows. The anti-GFP antibody only yielded one band, confirming the stability of the fusion protein. **(C)** Colocalization of GFP-Hop/Sti1 and an ER-mCherry marker in PVY^N^-infected (first and second row) and in non-infected *Nicotiana benthamiana* plants (third row). In PVY^N^-infected cells, GFP-Hop/Sti1 was found to be retained in aggregates particularly in the vicinity of the nucleus (panels I, nucleus marked with N, arrowheads point to aggregates) that colocalized with a coexpressed ER-marker (panels II, ER-mCherry marker; panels III, merged images, arrowheads point to aggregates). In non-infected cells, no such aggregates could be observed. Images are maximum projections of confocal Z-stacks, scale bars represent 10 μm.

The failure of PVY^N^ to benefit from the reduced host defense response in Hop/Sti1-silenced plants may be explained by a role of the cochaperone during viral replication/translation. In contrast to the rice blast fungus, which benefits from the silencing of Hop/Sti1 in rice (Chen et al., 2010), plant viruses are heavily dependent on miscellaneous host factors to ensure their own genome translation, replication, cell-to-cell movement, as well as other functions vital to effective establishment of virulence (Jiang et al., 2006; Stapleford and Miller, 2010; Heinlein, 2015). Assuming that PVY^N^ is relying on the cochaperone for its own proliferation, a reduced virus accumulation, despite a lower basal defense response of the host, seems conceivable. To investigate whether Hop/Sti1 may be associated with virus replication/translation, *Nicotiana tabacum* Hop/Sti1 was fused to the carboxy-terminus of the green fluorescent protein GFP for localization studies in *Nicotiana benthamiana*. Consistent with previous studies, the fusion protein was found in the cytoplasm and the ER (Chen et al., 2010; Popescu, 2012), but also in the nucleus (**Figure 9A**). Western blotting using both an anti-Hop/Sti1 antibody and an anti-GFP antibody confirmed the integrity of the construct, since no degradation products could be detected (**Figure 9B**). To ascertain whether Hop/Sti1 is involved in viral processes, we expressed the GFP-tagged protein in PVY^N^-infected and in non-infected *Nicotiana benthamiana*. Since it is well-known that potyviral infections lead to rearrangements of the ER-membrane system to facilitate viral replication and translation (Schaad et al., 1997; Cotton et al., 2009; Grangeon et al., 2012; Ivanov et al., 2014; Heinlein, 2015), an ER-marker fused to mCherry was coexpressed (Nelson et al., 2007). In PVY^N^-infected cells, ER-membranes stained by the marker protein appeared as aggregates and presumably represent aforementioned viral replication complexes (VRCs, **Figure 9C**, first and second row, panels II). Remarkably, coexpressed GFP-Hop/Sti1 is also recruited to the virus-induced aggregates (**Figure 9C**, first and second row, panels I), which colocalize with the ER-marker-stained VRCs (**Figure 9C**, first and second row, panels III). In contrast to this, such aggregates could never be observed in non-infected cells (**Figure 9C**, third row). This finding demonstrates that Hop/Sti1 changes its localization in virus infected cells, suggesting its involvement in viral replication and/or translation.

### Silencing of Hop/Sti1 in Tobacco Does Not Interfere with the Unfolded Protein Response

**FIGURE 10:**
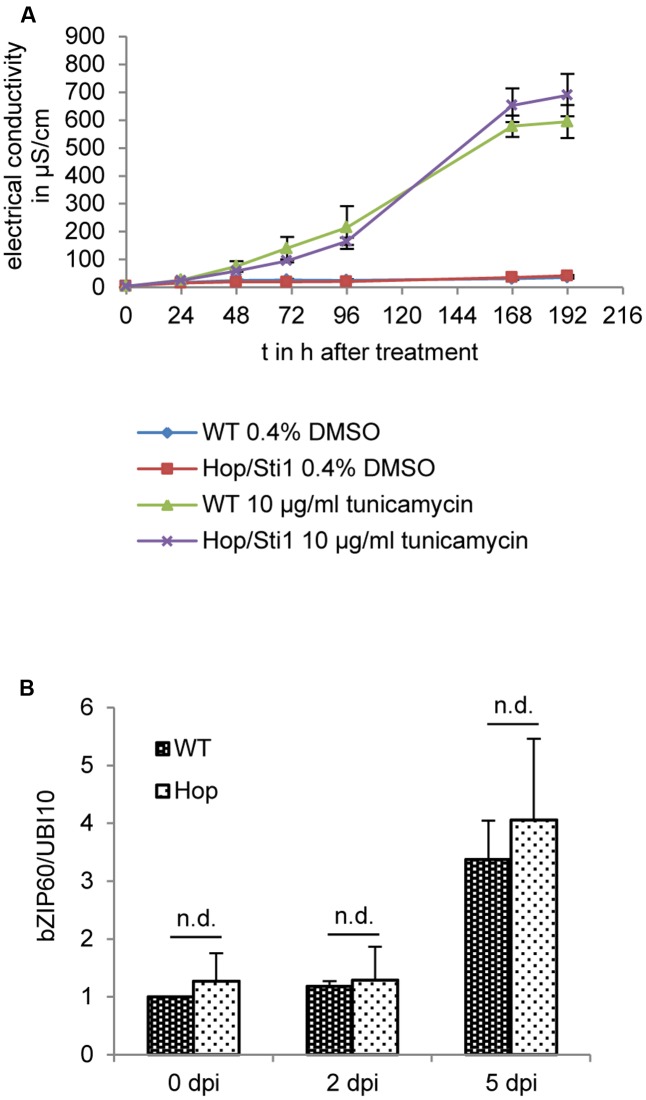
Hop/Sti1-knockdown does not affect the unfolded protein response (UPR) in *Nicotiana tabacum* leaves. **(A)** Cell death in response to tunicamycin treatment in wild-type and Hop/Sti1-RNAi plants. Leaf disks were floated on a tunicamycin-solution, ion leakage due to cell death was measured as electrical conductivity of the solution. Error bars represent the standard error. **(B)** Induction of the UPR by PVY^N^. Analysis of relative content of spliced bZIP60 by quantitative real-time PCR showed no significant difference (Bonferroni-corrected students *t*-test, *p* < 0.05) between wild-type and Hop/Sti1-RNAi plants. Samples were taken from inoculated leaves at the indicated time points. Error bars represent the standard error.

The UPR represents a cellular reaction to the accumulation of un- or misfolded proteins in the ER, aiming to restore protein homeostasis. Failure at this restoration ultimately leads to the induction of apoptosis or programmed cell death (Adamakis et al., 2011; Walter and Ron, 2011; Iwata and Koizumi, 2012; Williams et al., 2014). Of note, a recent study in *Arabidopsis thaliana* implicated a Hop/Sti1-homolog in the ER stress response, suggesting *At*HOP3 being a negative regulator of this pathway (Fernández-Bautista et al., 2017). In addition to this, other studies showed that potyviral infections also lead to an induction of the UPR. Here, the ER-membrane localized unfolded protein sensor inositol-requiring enzyme 1(IRE1) mediates the unconventional splicing of cytosolic bZIP60 mRNA, generating the template for an active transcription factor that can upregulate the expression of certain UPR target genes in the nucleus. However, it seems still unclear whether the virus benefits from this induction (Zhang et al., 2015) or whether the UPR poses a defense mechanism restricting viral spread (Gaguancela et al., 2016). In any case, considering the observed colocalization of Hop/Sti1 with aggregates evidently derived from the ER and the above-mentioned fact that prolonged ER stress may induce programmed cell death, we next investigated a possible connection of the ER stress pathway and the loss of PVY^N^ symptoms in Hop/Sti1-RNAi plants. To this end, we first inhibited N-linked protein glycosylation by treatment of either wild-type or Hop/Sti1-RNAi leaf disks with tunicamycin and thereby provoked accumulation of unfolded proteins (Takatsuki and Tamura, 1971; Tkacz and Lampen, 1975). As an indicator of cell death, we measured the electrical conductivity of the floating buffer medium caused by leakage of ions from the dying cells. During the observation period of 8 days, the electrical conductivity of the wild-type control plant samples rose to a value of approximately 600 μS cm^-1^, while untreated samples remained at a basal value of ca. 40 μS cm^-1^ (**Figure 10A**). Regarding the tunicamycin-treated samples derived from the transgenic line #61, we did not find significant differences to the wild-type samples. This suggests that programmed cell death in fully grown leaves, mediated by the tunicamycin-induced UPR, is not affected by the knockdown of the cochaperone Hop/Sti1. In order to not neglect viral processes and mechanisms, we furthermore investigated the UPR-induction in response to PVY^N^ infection in wild-type and Hop/Sti1-knockdown plants. To this end, total RNA was isolated from locally infected leaves prior to infection as well as 2 and 5 days after inoculation. Using quantitative real-time PCR, the relative content of spliced bZIP60 mRNA was determined as reported before (Gaguancela et al., 2016). In support of the tunicamycin-floating assay, we could find a similar increase in bZIP60-splicing over time for both wild-type and Hop/Sti1-RNAi samples (**Figure 10B** and as boxplot: Supplementary Figure S4). In summary, the knockdown of Hop/Sti1 by RNAi does not affect the induction of the UPR or the affiliated programmed cell death reaction in response to PVY^N^ or tunicamycin in *Nicotiana tabacum* leaves, i.e., the Hop/Sti1-dependent formation of PVY^N^ infection symptoms does not stem from the induction of ER stress-mediated cell death.

## Discussion

In this study, we investigated the role of the tobacco Hsp70/Hsp90 organizing protein Hop/Sti1 in maturation and trafficking of plant RLKs. The cochaperone has previously been shown to participate in the maturation and plasma membrane-targeting of rice chitin PRR CERK1, with which it interacts both at the ER and the plasma membrane. Rice plants defective of Hop/Sti1 could be shown to be more susceptible to rice blast fungus, whereas overexpressing lines were more resistant (Chen et al., 2010). Although it has been speculated that Hop/Sti1 plays a central role in PRR trafficking in general, PRRs other than CERK1 were not examined in depth. To test for a general function of Hop/Sti1 in PRR trafficking, we generated *Nicotiana tabacum* cv. Samsun NN plants stably expressing an RNAi construct to silence Hop/Sti1 expression. Similar to what has been observed in rice, the transgenic plants were not able to respond to CERK1-stimulation. Since the study in rice stated that Hop/Sti1 interacts with *Oryza sativa* FLS2 in yeast-based split-ubiquitin- and in bimolecular fluorescence complementation experiments, but no evaluation of a functional dependency was performed, we expressed a FLS2-mCherry fusion protein under control of the FLS2 promoter in wild-type and transgenic plants. Noteworthy, CERK1 belongs to the family of lysine-motif RLKs, while FLS2 is a member of the leucine-rich repeat RLK class (Shiu et al., 2004; Miya et al., 2007). Microscopical analyses revealed successful targeting of FLS2 to the plasma membrane in wild-type and transgenic plants, suggesting a Hop/Sti1-independent trafficking mechanism. To investigate the functional integrity of the PRR, leaf disks were furthermore treated with flg22 in a floating assay. Both wild-type and Hop/Sti1-silenced leaf disks were able to react to the stimulus with generation of ROS, proving that the receptor is still functional. In conclusion, this shows that Hop/Sti1 is not a generally required facilitator of PRR-plasma membrane transport, but specifically takes part in the maturation of CERK1. Regarding the previously published interaction between Hop/Sti1 and FLS2, a functional relevance could not be observed. Whether other PRRs are also dependent on Hop/Sti1 for correct trafficking remains to be investigated in future studies.

In general, immune responses to virus infections are mounted by recognition of viral proteins by R proteins (Mandadi and Scholthof, 2013). In case of the well-studied interaction between tobacco mosaic virus (TMV) and the R protein N, the viral replicase p50 interacts with the N-receptor interacting protein 1 (NRIP1) in the cytoplasm. This protein complex is recognized by the N immune receptor, which then induces defense signaling, eventually mediating TMV-resistance (Caplan et al., 2008). Intriguingly, efficient N-mediated TMV-resistance is also dependent on the presence of a RLK of unknown function, designated induced RLK (IRK), whose maturation is dependent on ER-localized chaperones such as protein disulfide isomerases and calreticulins (Caplan et al., 2009). This suggests that recognition and/or establishment of anti-viral defense responses is not the sole domain of R proteins, but also involves plasma membrane-localized receptors. Hence, we aimed to gain further insight into a potential role of Hop/Sti1 during plant virus perception. Therefore, the transgenic lines were inoculated with PVY^N^, leading to a loss of infection symptoms, but only a moderate reduction of PVY^N^ titer and no impairment of virus progeny infectivity. Although the origin of viral symptom development is not entirely understood, evidence exist that, e.g., cell death, as seen during systemic necrosis, is a consequence of a host immune response failing to ultimately restrict viral spread (Komatsu et al., 2010; Pacheco et al., 2012). Considering this, we hypothesized that, due to a defect in PRR/RLK-maturation/trafficking, Hop/Sti1-RNAi plants were not able to perceive the viral presence anymore, and thus failed to induce defense-related reactions. Indeed, infected transgenic plants showed decreased PR-gene expression and lower SA-levels compared to wild-type, supporting the proposition of a connection of plant defense responses and viral symptom development. Furthermore, it strengthens the assumption of an involvement of Hop/Sti1 in PVY^N^ recognition, possibly during receptor maturation or trafficking.

The observed impairment of basal immune responses in Hop/Sti1-RNAi plants seems to be in conflict with the slightly reduced PVY^N^ titer. In fact, one would rather expect an increase in viral proliferation, since PVY^N^ is not facing any resistance anymore. However, the single-stranded RNA genome of PVY encodes only 11 viral proteins, and thus is well-known to be heavily dependent on co-opting cellular proteins (Urcuqui-Inchima et al., 2001; Chung et al., 2008). Especially chaperone proteins of host plants are crucial for several steps during the potyvirus replication cycle (reviewed by Verchot, 2012). For instance, the proteasomal degradation of viral capsid protein during viral RNA uncoating is mediated by delivery of capsid protein to Hsp70 by cochaperone Hsp40, where it is designated for degradation by polyubiquitination (Hofius et al., 2007; Hafren et al., 2010). Furthermore, the viral RNA-dependent RNA polymerase was found to interact with an Hsp70 protein in ER-derived membranes, possibly representing viral replication complexes (Dufresne et al., 2008). Therefore, an involvement of the cochaperone in a virus-related process seemed conceivable. In agreement with this, GFP-tagged Hop/Sti1 was found in aggregates in PVY^N^-infected *N. benthamiana* cells, while this was not the case in non-infected plants. Coexpression with an ER-marker revealed that the observed aggregates colocalize with aggregates derived from the ER, and therefore may represent VRCs. Thus, it is tempting to speculate about a certain function of Hop/Sti1 in optimization of viral replication, and the aforementioned presence of Hop/Sti1 interactor Hsp70 in the same complex endorses this hypothesis even more.

It was only recently shown that potyvirus infection induces ER stress and the UPR (Zhang et al., 2015; Gaguancela et al., 2016). Since prolonged ER stress also leads to programmed cell death (Adamakis et al., 2011; Walter and Ron, 2011; Iwata and Koizumi, 2012; Williams et al., 2014) and *At*HOP3 was implicated in negative regulation of the ER stress response (Fernández-Bautista et al., 2017), we assessed the relation of Hop/Sti1, the UPR and PVY infection in *Nicotiana tabacum*. However, Hop/Sti1-RNAi plants showed no significant difference in tunicamycin-induced cell death or PVY^N^ infection-mediated splicing of bZIP60. Hence, in contrast to the null-mutant utilized in the study in *Arabidopsis thaliana* mentioned above, the mere knockdown of the cochaperone by RNAi does neither enhance, nor impair ER stress-induced cell death in *Nicotiana tabacum* leaves. Furthermore, PVY^N^-induced symptoms are not a consequence of prolonged ER stress leading to cell death, but occur rather independent of the UPR pathway.

In summary, the cochaperone Hop/Sti1 is a pivotal cellular component for maturation and plasma membrane trafficking of certain, but not all PRRs/RLKs. Future studies will have to elucidate, to what extent PRRs/RLKs other than CERK1 are relying on the same pathway. We furthermore hypothesize that, during infection with PVY^N^, Hop/Sti1 is playing an ambivalent role: On the one hand, it is recruited by the viral machinery to boost PVY proliferation, possibly at the stage of viral genome replication. On the other hand, the cochaperone is necessary to enable sensing of the viral presence in the host and thus allowing for induction of defense responses. This may be achieved by a yet unknown PRR/RLK, which is contributing to antiviral measures similar to IRK in N-mediated TMV resistance. In the interaction of PVY^N^ with *Nicotiana tabacum*, however, these antiviral measures fail to restrict the virus and are thus perceived as necrotic symptoms. Should this hypothesis prove true, future studies of Hop/Sti1’s target receptors will lead to a better understanding of virus-host interactions, antiviral defense responses and possibly plant innate immunity in general.

## Author Contributions

US and H-PM conceived and designed the experiments; JH measured extracted SA by HPLC; H-PM and FB generated the Hop/Sti1-RNAi-line and antibody; CL and MK performed the experiments and together with US analyzed the data; CL and US wrote the manuscript.

## Conflict of Interest Statement

The authors declare that the research was conducted in the absence of any commercial or financial relationships that could be construed as a potential conflict of interest.

## Footnotes

1 https://abrc.osu.edu/
