# Supplementary material for: Hop/Sti1 – A Two-Faced Cochaperone Involved in Pattern Recognition Receptor Maturation and Viral Infection
Source: Front Plant Sci. 2017 Oct 11;8:1754. doi: 10.3389/fpls.2017.01754 (PMC5641557; doi:10.3389/fpls.2017.01754)
Supplement: Supplementary file 1 [file Data_Sheet_1.DOCX]

Supplementary Material

Hop/Sti1 – A two-faced cochaperone involved in Pattern Recognition Receptor maturation and viral infection

Christian E. Lamm, Max. E. Kraner, Jörg Hofmann, Frederik Börnke, Hans-Peter Mock, Uwe Sonnewald*

*** Correspondence:** Uwe Sonnewald: [Uwe.Sonnewald@FAU.de](mailto:Uwe.Sonnewald@FAU.de)

# Supplementary Figures


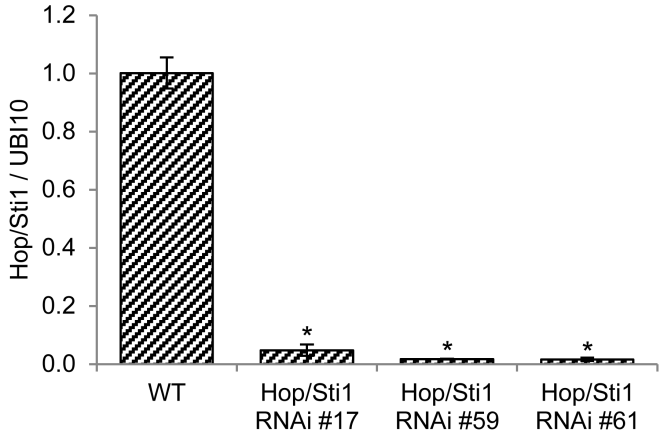


Supplementary Figure 1: Hop/Sti1 expression was analyzed by quantitative real-time PCR. Expression is significantly reduced in Hop/Sti1-RNAi lines compared to wild-type plants (Student’s t-test, p<0.05).


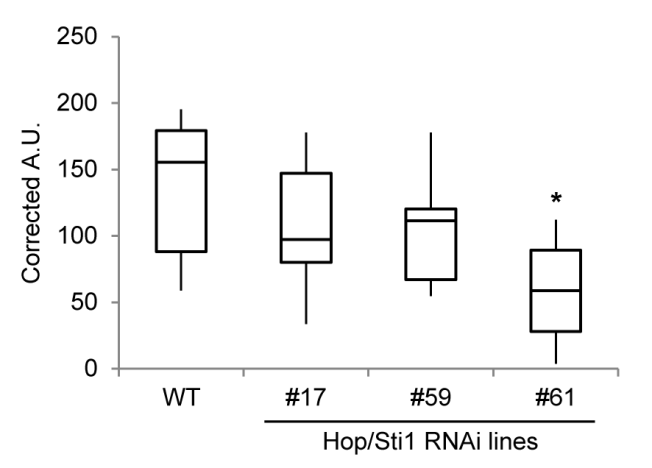


**Supplementary Figure 2:** Boxplot PVY-CP ELISA. PVY^N^ titer was determined in infected wild-type and Hop/Sti1-RNAi plants. An asterisk indicates significance (Bonferroni-corrected student’s t-test, p-value: 1.89E-02<0.05).

**
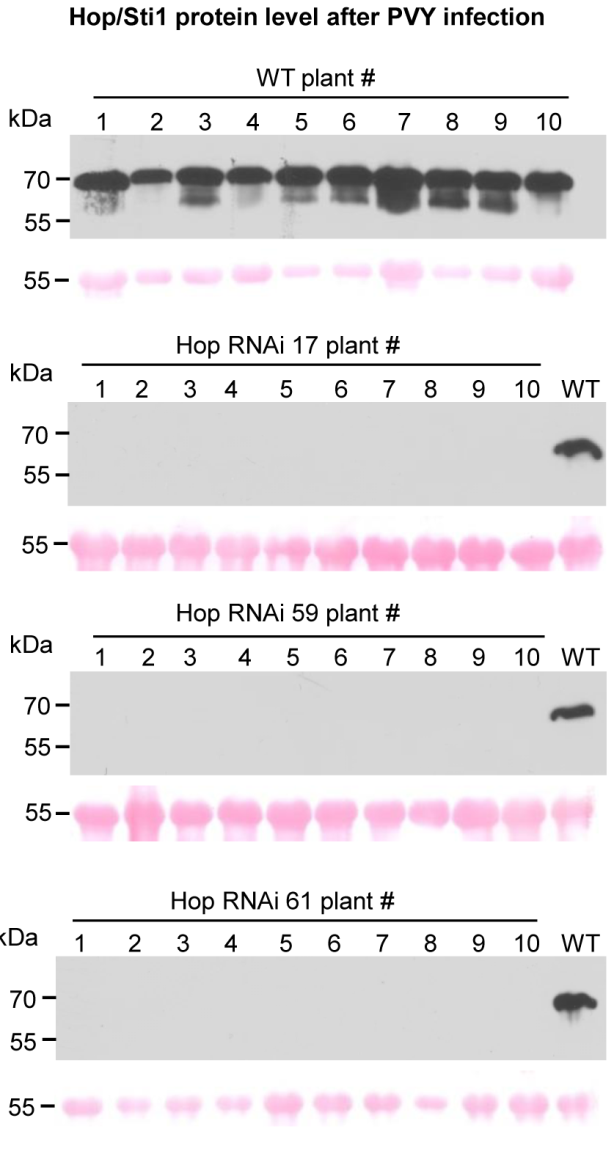
**

**Supplementary Figure 3.** Hop/Sti1 protein level after PVY infection, determined by Western blotting. Ten plants of each individual transgenic line as well as wild-type plants were sampled twelve days after PVY^N^ inoculation and subjected to standard Western blotting. Detection was carried out using a Hop/Sti1-specific antibody, Ponceau S staining as loading control. While all wild-type plants exhibited strong expression of the cochaperone, the protein was hardly detectable in RNAi-lines, confirming the silencing of Hop/Sti1 also in infected leaf tissue.


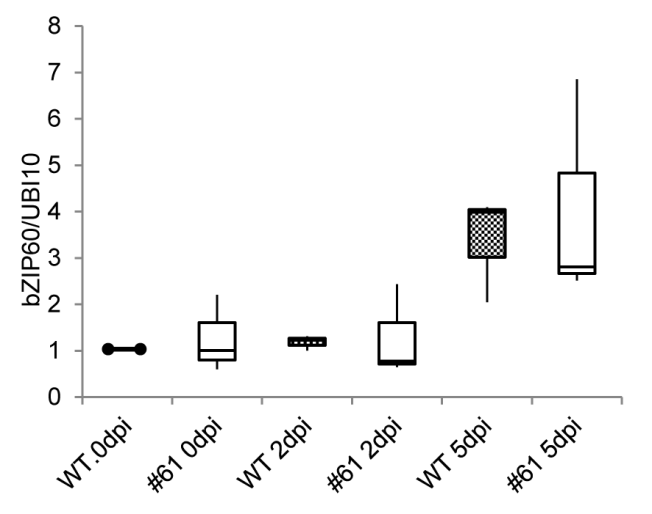


**Supplementary Figure S4:** Boxplot qPCR bZiP60s. Analysis of relative content of spliced bZIP60 by quantitative real-time PCR showed no significant difference (Bonferroni-corrected students t-test, p<0.05) between wild-type and Hop/Sti1-RNAi plants. 0 dpi wild-type values set to 1, samples were taken from inoculated leaves at the indicated time points.

# Supplementary Tables

| **Multiple Comparisons relative PR-1 expression** | | | | | | |
| --- | --- | --- | --- | --- | --- | --- |
| Dependent Variable: | PR1_Expression |  |  |  |  |  |
| Tukey HSD |  |  |  |  |  |  |
| (I) Group | | Mean Difference (I-J) | Std. Error | Sig. | 95% Confidence Interval | |
|  |  |  |  |  | Lower Bound | Upper Bound |
| WT -PVY | WT +PVY | -64,06621^*^ | 6.38840 | 1.2E-05 | -85.0910 | -43.0415 |
|  | HopRNAi #17 +PVY | -21,19786^*^ | 6.38840 | 4.8E-02 | -42.2226 | -0.1731 |
|  | HopRNAi #59 +PVY | -9.29485 | 6.38840 | 6.1E-01 | -30.3196 | 11.7299 |
|  | HopRNAi #61 +PVY | -4.25417 | 6.38840 | 9.6E-01 | -25.2789 | 16.7706 |
| WT +PVY | WT -PVY | 64,06621^*^ | 6.38840 | 1.2E-05 | 43.0415 | 85.0910 |
|  | HopRNAi #17 +PVY | 42,86835^*^ | 6.38840 | 3.9E-04 | 21.8436 | 63.8931 |
|  | HopRNAi #59 +PVY | 54,77137^*^ | 6.38840 | 4.9E-05 | 33.7466 | 75.7961 |
|  | HopRNAi #61 +PVY | 59,81205^*^ | 6.38840 | 2.2E-05 | 38.7873 | 80.8368 |
| HopRNAi #17 +PVY | WT -PVY | 21,19786^*^ | 6.38840 | 4.8E-02 | 0.1731 | 42.2226 |
|  | WT +PVY | -42,86835^*^ | 6.38840 | 3.9E-04 | -63.8931 | -21.8436 |
|  | HopRNAi #59 +PVY | 11.90301 | 6.38840 | 3.9E-01 | -9.1217 | 32.9278 |
|  | HopRNAi #61 +PVY | 16.94369 | 6.38840 | 1.3E-01 | -4.0811 | 37.9684 |
| HopRNAi #59 +PVY | WT -PVY | 9.29485 | 6.38840 | 6.1E-01 | -11.7299 | 30.3196 |
|  | WT +PVY | -54,77137^*^ | 6.38840 | 4.9E-05 | -75.7961 | -33.7466 |
|  | HopRNAi #17 +PVY | -11.90301 | 6.38840 | 3.9E-01 | -32.9278 | 9.1217 |
|  | HopRNAi #61 +PVY | 5.04068 | 6.38840 | 9.3E-01 | -15.9841 | 26.0654 |
| HopRNAi #61 +PVY | WT -PVY | 4.25417 | 6.38840 | 9.6E-01 | -16.7706 | 25.2789 |
|  | WT +PVY | -59,81205^*^ | 6.38840 | 2.2E-05 | -80.8368 | -38.7873 |
|  | HopRNAi #17 +PVY | -16.94369 | 6.38840 | 1.3E-01 | -37.9684 | 4.0811 |
|  | HopRNAi #59 +PVY | -5.04068 | 6.38840 | 9.3E-01 | -26.0654 | 15.9841 |
| *. The mean difference is significant at the 0.05 level. | | | | | | |

**Supplementary Table 1:** PR-1 qPCR, analysis of variance with post-hoc Tukey HSD. The table depicts the mean difference between tested groups, the standard error (Std. Error), the p-value (Sig.) and the 95% confidence intervals with lower and upper bounds. A value of 0.05 was used as significance level.

| **Multiple Comparisons relative PR-2 expression** | | | | | | |
| --- | --- | --- | --- | --- | --- | --- |
| Dependent Variable: | PR2_Expression |  |  |  |  |  |
| Tukey HSD |  |  |  |  |  |  |
| (I) Group | | Mean Difference (I-J) | Std. Error | Sig. | 95% Confidence Interval | |
|  |  |  |  |  | Lower Bound | Upper Bound |
| WT -PVY | WT +PVY | -3,23465^*^ | 0.37199 | 4.3E-05 | -4.4589 | -2.0104 |
|  | HopRNAi #17 +PVY | -1,27679^*^ | 0.37199 | 4.0E-02 | -2.5010 | -0.0525 |
|  | HopRNAi #59 +PVY | -1.08840 | 0.37199 | 8.8E-02 | -2.3126 | 0.1358 |
|  | HopRNAi #61 +PVY | -1,53909^*^ | 0.37199 | 1.4E-02 | -2.7633 | -0.3149 |
| WT +PVY | WT -PVY | 3,23465^*^ | 0.37199 | 4.3E-05 | 2.0104 | 4.4589 |
|  | HopRNAi #17 +PVY | 1,95786^*^ | 0.37199 | 2.6E-03 | 0.7336 | 3.1821 |
|  | HopRNAi #59 +PVY | 2,14625^*^ | 0.37199 | 1.3E-03 | 0.9220 | 3.3705 |
|  | HopRNAi #61 +PVY | 1,69556^*^ | 0.37199 | 7.2E-03 | 0.4713 | 2.9198 |
| HopRNAi #17 +PVY | WT -PVY | 1,27679^*^ | 0.37199 | 4.0E-02 | 0.0525 | 2.5010 |
|  | WT +PVY | -1,95786^*^ | 0.37199 | 2.6E-03 | -3.1821 | -0.7336 |
|  | HopRNAi #59 +PVY | 0.18838 | 0.37199 | 9.8E-01 | -1.0359 | 1.4126 |
|  | HopRNAi #61 +PVY | -0.26230 | 0.37199 | 9.5E-01 | -1.4865 | 0.9619 |
| HopRNAi #59 +PVY | WT -PVY | 1.08840 | 0.37199 | 8.8E-02 | -0.1358 | 2.3126 |
|  | WT +PVY | -2,14625^*^ | 0.37199 | 1.3E-03 | -3.3705 | -0.9220 |
|  | HopRNAi #17 +PVY | -0.18838 | 0.37199 | 9.8E-01 | -1.4126 | 1.0359 |
|  | HopRNAi #61 +PVY | -0.45069 | 0.37199 | 7.5E-01 | -1.6749 | 0.7736 |
| HopRNAi #61 +PVY | WT -PVY | 1,53909^*^ | 0.37199 | 1.4E-02 | 0.3149 | 2.7633 |
|  | WT +PVY | -1,69556^*^ | 0.37199 | 7.2E-03 | -2.9198 | -0.4713 |
|  | HopRNAi #17 +PVY | 0.26230 | 0.37199 | 9.5E-01 | -0.9619 | 1.4865 |
|  | HopRNAi #59 +PVY | 0.45069 | 0.37199 | 7.5E-01 | -0.7736 | 1.6749 |
| *. The mean difference is significant at the 0.05 level. | | | | | | |

**Supplementary Table 2:** PR-2 qPCR, analysis of variance with post-hoc Tukey HSD. The table depicts the mean difference between tested groups, the standard error (Std. Error), the p-value (Sig.) and the 95% confidence intervals with lower and upper bounds. A value of 0.05 was used as significance level.

| **Multiple Comparisons SA content** | | | | | | |
| --- | --- | --- | --- | --- | --- | --- |
| Dependent Variable: | SA_Content |  |  |  |  |  |
| Tukey HSD |  |  |  |  |  |  |
| (I) Group | | Mean Difference (I-J) | Std. Error | Sig. | 95% Confidence Interval | |
|  |  |  |  |  | Lower Bound | Upper Bound |
| WT 0dpi | WT 5dpi | 5.85546 | 24.01420 | 1.0E+00 | -76.0645 | 87.7754 |
|  | WT 12dpi 3. leaf | -120,57596^*^ | 24.01420 | 1.8E-03 | -202.4959 | -38.6561 |
|  | WT 12dpi 5. leaf | -462,38980^*^ | 22.23283 | 2.3E-12 | -538.2329 | -386.5467 |
|  | hop RNAi #61 0dpi | 10.24307 | 24.01420 | 1.0E+00 | -71.6768 | 92.1630 |
|  | hop RNAi #61 5dpi | -9.07609 | 24.01420 | 1.0E+00 | -90.9960 | 72.8438 |
|  | hop RNAi #61 12dpi 3. leaf | -1.48046 | 24.01420 | 1.0E+00 | -83.4004 | 80.4394 |
|  | hop RNAi #61 12dpi 5. leaf | -248,42654^*^ | 24.01420 | 1.3E-07 | -330.3464 | -166.5066 |
| WT 5dpi | WT 0dpi | -5.85546 | 24.01420 | 1.0E+00 | -87.7754 | 76.0645 |
|  | WT 12dpi 3. leaf | -126,43142^*^ | 25.67226 | 2.2E-03 | -214.0075 | -38.8554 |
|  | WT 12dpi 5. leaf | -468,24526^*^ | 24.01420 | 4.8E-12 | -550.1652 | -386.3254 |
|  | hop RNAi #61 0dpi | 4.38761 | 25.67226 | 1.0E+00 | -83.1885 | 91.9637 |
|  | hop RNAi #61 5dpi | -14.93155 | 25.67226 | 1.0E+00 | -102.5076 | 72.6445 |
|  | hop RNAi #61 12dpi 3. leaf | -7.33592 | 25.67226 | 1.0E+00 | -94.9120 | 80.2401 |
|  | hop RNAi #61 12dpi 5. leaf | -254,28199^*^ | 25.67226 | 2.5E-07 | -341.8581 | -166.7059 |
| WT 12dpi 3. leaf | WT 0dpi | 120,57596^*^ | 24.01420 | 1.8E-03 | 38.6561 | 202.4959 |
|  | WT 5dpi | 126,43142^*^ | 25.67226 | 2.2E-03 | 38.8554 | 214.0075 |
|  | WT 12dpi 5. leaf | -341,81384^*^ | 24.01420 | 7.6E-10 | -423.7338 | -259.8939 |
|  | hop RNAi #61 0dpi | 130,81903^*^ | 25.67226 | 1.6E-03 | 43.2430 | 218.3951 |
|  | hop RNAi #61 5dpi | 111,49987^*^ | 25.67226 | 7.5E-03 | 23.9238 | 199.0759 |
|  | hop RNAi #61 12dpi 3. leaf | 119,09550^*^ | 25.67226 | 4.0E-03 | 31.5194 | 206.6716 |
|  | hop RNAi #61 12dpi 5. leaf | -127,85058^*^ | 25.67226 | 2.0E-03 | -215.4266 | -40.2745 |
| WT 12dpi 5. leaf | WT 0dpi | 462,38980^*^ | 22.23283 | 2.3E-12 | 386.5467 | 538.2329 |
|  | WT 5dpi | 468,24526^*^ | 24.01420 | 4.8E-12 | 386.3254 | 550.1652 |
|  | WT 12dpi 3. leaf | 341,81384^*^ | 24.01420 | 7.6E-10 | 259.8939 | 423.7338 |
|  | hop RNAi #61 0dpi | 472,63287^*^ | 24.01420 | 4.2E-12 | 390.7130 | 554.5528 |
|  | hop RNAi #61 5dpi | 453,31371^*^ | 24.01420 | 7.5E-12 | 371.3938 | 535.2336 |
|  | hop RNAi #61 12dpi 3. leaf | 460,90934^*^ | 24.01420 | 5.9E-12 | 378.9894 | 542.8293 |
|  | hop RNAi #61 12dpi 5. leaf | 213,96327^*^ | 24.01420 | 1.2E-06 | 132.0434 | 295.8832 |
| hop RNAi #61 0dpi | WT 0dpi | -10.24307 | 24.01420 | 1.0E+00 | -92.1630 | 71.6768 |
|  | WT 5dpi | -4.38761 | 25.67226 | 1.0E+00 | -91.9637 | 83.1885 |
|  | WT 12dpi 3. leaf | -130,81903^*^ | 25.67226 | 1.6E-03 | -218.3951 | -43.2430 |
|  | WT 12dpi 5. leaf | -472,63287^*^ | 24.01420 | 4.2E-12 | -554.5528 | -390.7130 |
|  | hop RNAi #61 5dpi | -19.31916 | 25.67226 | 9.9E-01 | -106.8952 | 68.2569 |
|  | hop RNAi #61 12dpi 3. leaf | -11.72353 | 25.67226 | 1.0E+00 | -99.2996 | 75.8525 |
|  | hop RNAi #61 12dpi 5. leaf | -258,66961^*^ | 25.67226 | 1.9E-07 | -346.2457 | -171.0935 |
| hop RNAi #61 5dpi | WT 0dpi | 9.07609 | 24.01420 | 1.0E+00 | -72.8438 | 90.9960 |
|  | WT 5dpi | 14.93155 | 25.67226 | 1.0E+00 | -72.6445 | 102.5076 |
|  | WT 12dpi 3. leaf | -111,49987^*^ | 25.67226 | 7.5E-03 | -199.0759 | -23.9238 |
|  | WT 12dpi 5. leaf | -453,31371^*^ | 24.01420 | 7.5E-12 | -535.2336 | -371.3938 |
|  | hop RNAi #61 0dpi | 19.31916 | 25.67226 | 9.9E-01 | -68.2569 | 106.8952 |
|  | hop RNAi #61 12dpi 3. leaf | 7.59563 | 25.67226 | 1.0E+00 | -79.9804 | 95.1717 |
|  | hop RNAi #61 12dpi 5. leaf | -239,35044^*^ | 25.67226 | 6.2E-07 | -326.9265 | -151.7744 |
| hop RNAi #61 12dpi 3. leaf | WT 0dpi | 1.48046 | 24.01420 | 1.0E+00 | -80.4394 | 83.4004 |
|  | WT 5dpi | 7.33592 | 25.67226 | 1.0E+00 | -80.2401 | 94.9120 |
|  | WT 12dpi 3. leaf | -119,09550^*^ | 25.67226 | 4.0E-03 | -206.6716 | -31.5194 |
|  | WT 12dpi 5. leaf | -460,90934^*^ | 24.01420 | 5.9E-12 | -542.8293 | -378.9894 |
|  | hop RNAi #61 0dpi | 11.72353 | 25.67226 | 1.0E+00 | -75.8525 | 99.2996 |
|  | hop RNAi #61 5dpi | -7.59563 | 25.67226 | 1.0E+00 | -95.1717 | 79.9804 |
|  | hop RNAi #61 12dpi 5. leaf | -246,94608^*^ | 25.67226 | 3.9E-07 | -334.5221 | -159.3700 |
| hop RNAi #61 12dpi 5. leaf | WT 0dpi | 248,42654^*^ | 24.01420 | 1.3E-07 | 166.5066 | 330.3464 |
|  | WT 5dpi | 254,28199^*^ | 25.67226 | 2.5E-07 | 166.7059 | 341.8581 |
|  | WT 12dpi 3. leaf | 127,85058^*^ | 25.67226 | 2.0E-03 | 40.2745 | 215.4266 |
|  | WT 12dpi 5. leaf | -213,96327^*^ | 24.01420 | 1.2E-06 | -295.8832 | -132.0434 |
|  | hop RNAi #61 0dpi | 258,66961^*^ | 25.67226 | 1.9E-07 | 171.0935 | 346.2457 |
|  | hop RNAi #61 5dpi | 239,35044^*^ | 25.67226 | 6.2E-07 | 151.7744 | 326.9265 |
|  | hop RNAi #61 12dpi 3. leaf | 246,94608^*^ | 25.67226 | 3.9E-07 | 159.3700 | 334.5221 |
| *. The mean difference is significant at the 0.05 level. | | | | | | |

**Supplementary Table 3:** Salicylic acid content, analysis of variance with post-hoc Tukey HSD. The table depicts the mean difference between tested groups, the standard error (Std. Error), the p-value (Sig.) and the 95% confidence intervals with lower and upper bounds. A value of 0.05 was used as significance level.
